# Traditional knowledge on wild and cultivated plants in the Kilombero Valley (Morogoro Region, Tanzania)

**DOI:** 10.1186/s13002-017-0146-y

**Published:** 2017-03-09

**Authors:** Mirko Salinitro, Renzo Vicentini, Costantino Bonomi, Annalisa Tassoni

**Affiliations:** 1 Department of Biological, Geological and Environmental Sciences, University of Bologna, via Irnerio 42, 40126 Bologna, Italy; 2 MUSE - Museo delle Scienze, Corso del Lavoro e della Scienza, 3, 38122 Trento, Italy

**Keywords:** Ethnobotany, Ethnomedicine, Medicinal plants, Udzungwa Mountains National Park, Kilombero Valley

## Abstract

**Background:**

This research was performed in four villages adjacent the boundary of Udzungwa Mountains National Park in the Kilombero River plain of Tanzania. The area adjacent the villages is characterized by self-consumption agriculture, with a population that is on average poor, still very tied to traditions and almost entirely unaffected by modernization and technology. The aim of the present study was to investigate and record local knowledge regarding the use of wild and traditionally cultivated plants used for traditional medicine and for other everyday purposes (e.g., food, fibers and timber).

**Methods:**

Ten traditional local healers, with solid botanical knowledge, were interviewed between June and August 2014 by means of semi-structured questionnaires. For each mentioned plant species, the Swahili folk name and, when possible, the classification by family, genus and species was recorded as well as the part of the plant used, the preparation method and the main uses (medicine, food or others).

**Results:**

In total 196 species were mentioned of which 118 could be botanically classified. The identified species belong to 44 different botanical families, with that of the Leguminosae being the most representative (24 species). The plants were mostly used as medical treatments (33.3% of the species) and foods (36.8%), and to produce wood and fibers (19.4%).

**Conclusion:**

The present study revealed that numerous plant species are still essential in the everyday life of the tribes living in Kilombero Valley. Most of the plants were usually harvested in the wild, however, after the creation of the Udzungwa Mountains National Park, the harvesting pressure has become concentrated on a few unprotected forest patches. Consequently, many useful species are becoming increasingly rare with the risk of losing the connected botanical and traditional knowledge. The present study may, therefore, contribute to record the ethnobotanical knowledge held by these populations, in order to preserve this valuable richness for future generations.

**Electronic supplementary material:**

The online version of this article (doi:10.1186/s13002-017-0146-y) contains supplementary material, which is available to authorized users.

## Background

Synthetic materials replaced nowadays many traditional plant-derived products having an increasing impact on the ethnobotanical culture of traditional societies. However, both wild and cultivated plants still remain vital to many aspects of traditional life [1]. In particular, plant species provide humans many type of building materials such as timber, poles and fibers [1–3]. Timber, the major forest product, has a considerable importance in the construction of temporary shelters, permanent homesteads and fences within the traditional societies [1], stems and leaves of grasses and palms are used in roof covering [2]. Plant parts have also additional uses in traditional arts and handicrafts including tool handles, cooking utensils, baskets, cordage and textiles [1, 4]. Likewise, plant extracts are sources of dyes, gums, latex, waxes, resins and adhesives [1, 2]. The most important uses of plants in developing countries (such as Tanzania) are however for fuel and medicine [5, 6].

In Tanzania, about 69% of the population lives in rural areas [7] where forest resources are central to their livelihood. Furthermore, according to the World Health Organisation (WHO), up to 80% of the population in developing countries depend on locally available plant resources for their primary healthcare [8]. In Tanzania, traditional medicine provides health care and support to over 60% of the rural population [9, 10]. This trend is mainly due to the strong attachment to traditions and spirituality and to the greater access, with respect to conventional medicine, to healers inside villages that provide low cost treatments [11]. The traditional medical treatments are mainly based on herbal remedies, using sometimes many different species mixed together [4, 12]. Except for a few plant species that grow inside or close to the villages, most of the used species are collected in forest areas [13].

Almost all the remaining forests in Tanzania are now found in protected areas, but in a few locations, with lower human population density, some unprotected forest patches [14] still remain. Public forest lands are freely available for use, whereas the exploitation of natural reserves is restricted by the issuing of licenses. Most of the unprotected forest land comprises *miombo* woodland (by the Swahili name of the common genus *Brachystegia*) which provides food, fuel, construction materials and medicines but is seriously threatened by overexploitation [15].

**Fig. 1:**
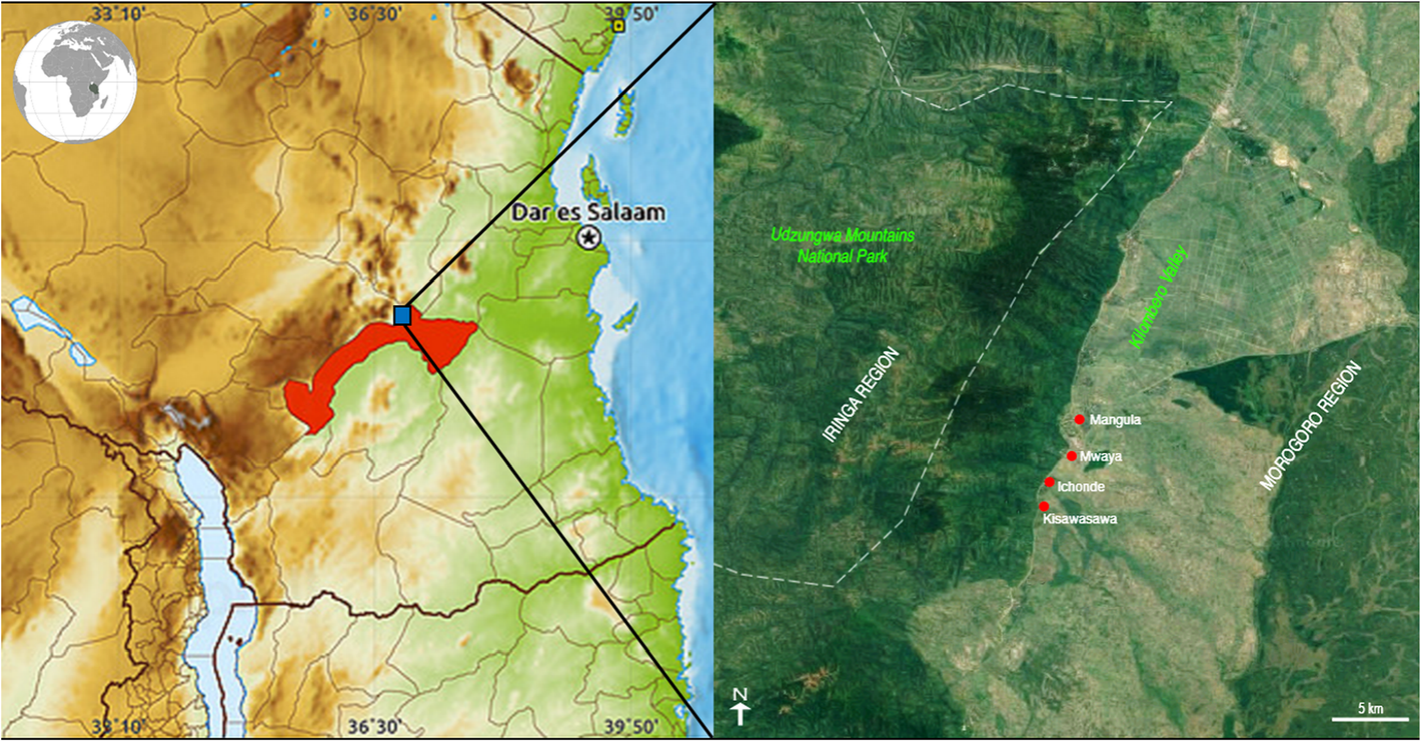
Study area. The enlargement shows the location of the four villages where the interviews took place

In Kilombero Valley (Morogoro region, where the present study was located, Fig. 1) deforestation was caused by the need to obtain land for agriculture and for the establishment of teak (*Tectona grandis* L.f.) and eucalyptus (*Eucalyptus globulus* Labill) plantations, as well as by charcoal production from cutted trees. Nonetheless the remaining lowland forest are still not protected unlike the neighbouring Udzungwa Mountains where a strong protection is carried out on the entire elevational range of the forest [14].

For years, local healers could bypass the restrictions for access to National Parks, but given the increasingly strict rules, they have lately been forced to change their places of collection with a serious impact on everyday life. In fact, the knowledge and experience of each traditional healer are deeply linked to the place where he/she learned and practiced plant collection over the years. There are now few forest areas in Kilombero Valley that can provide therapeutic plants. These are located far from the villages, and some of the collection methods, such as decortication, could be extremely impactful when carried out in small areas, making the plants unusable after a few years.

Since the founding of Udzungwa Mountains National Park, more than 24 years ago, there has been a depletion of the traditional medical culture, due to the forced abbandonement of familiar areas of collection, as well as the progressively more difficult transmission of knowledge to and training of young healers. Finally, the cost of traditional medicine is now starting to grow, causing a significant problem for people who have always relied on this method for their healthcare [11]. Since the 1970s, international health policy began to take interest in traditional medicine in Tanzania [11]; with the Traditional and Alternative Healthcare Practice Act 2002, the government recognizes traditional medicine as being important in the healthcare of its people. However, despite legislation being in place, not much progress has been made in the documentation and evaluation of the vast resource of medicinal plants used by traditional healers [16] and no actions have been taken to solve problems related to plant gathering practices.

Previous ethnobotanical studies have been conducted in Tanzania with the main purpose to investigate ethnomedicinal knowledge of local healers [9, 12, 13, 16–18], but only few have been carried out in the Morogoro region [5, 19, 20]. Some studies were also aimed at obtaining general ethnobotanical knowledge, including plant uses other than medicinal ones [5, 21].

The present study was carried out in four villages located between the agricultural area of Kilombero Valley and Udzungwa Mountains National Park (Morogoro region) (Fig. 1), in which the people obtain most of the raw materials for every day life and most medicinal plants from the forest environment [19]. Until 1992, year of the establishment of the Udzungwa Mountains National Park [22], the inhabitants of the area had free access to the forest and were able to dispose of its resources in an easy and sometimes indiscriminate way. Successively, restrictions have been introduced to limit the collection of plants inside the park, until the complete ban since 2011. Despite the efforts, made by the Authority managing the park and by non-profit organizations to educate the inhabitants of neighbouring villages to become independent from the forest, the total prohibition of using the park had a profound effect on people’s lifestyle and most of the traditional medicine practices.

The aim of the present research was to collect data about plants, both cultivated and wild, used for traditional medicine and for other everyday purposes (e.g., food, fibers, timber), in order to preserve an important endangered part of the local cultural heritage. In fact, if in general ethnobotanical knowledge is being progressively lost all over Africa due to modernization and globalization, this is even more so in the Udzungwa Mountains, where all the activities closely related to the use of plant natural resources are limited by the strict rules and regulations of the National Park.

## Methods

### Study area

The study was carried out in four villages (Mangula, Muaya, Ichonde, Kisawasawa), located close to the southern limit of the Udzungwa Mountains National Park (Morogoro region, Tanzania), in the large agricultural plain of the Kilombero river (Fig. 1). The study area, is dominated by a dry climate, with 750 mm of average annual rainfall. The climate is defined bi-seasonal and the annual variations are determined by the monsoons coming from the Indian Ocean. The hottest period starts in December and lasts until March. During this period, rainfall is abundant and the average maximum temperature is in December around 26.9 °C. The rain season reaches its peak in April. The dry and fresh season begins in May and lasts until October. The cooler months are June and July with an average temperature of 21.5 °C (https://it.climate-data.org/location/3040/). The vegetation of the area is mainly represented by Miombo woodlands, a subtropical formation typical of semi-arid and arid climate. Miombo is the Swahili word for the *Brachystegia* genus, which is the dominant tree in this natural environment together with *Julbernardia* and *Isoberlinia*, of the subfamily *Caesalpinioideae*. Artificial grassland and shrublands are also present due to human activities [23].

The lack of industrialization, the remoteness from urban centers, the proximity to mountains and the generalised poverty make this location a perfect nursery for the growth and preservation of ethnobotanical knowledge. In fact, in this area the effects of globalization are still very weak and the people are usually closely tied to their traditions.

In these communities, agriculture is the main economic activity, together with some animal husbandry often finalized to self-consumption, and the cultivated species are not numerous [5, 6]. Beside rice and corn, many other vegetables are grown, mostly belonging to the Solanaceae, Brassicaceae, Amaranthaceae and Cucurbitaceae families. Fruit trees, such as *Prunus persica* (L.) Batsch, *Persea americana* Mill., *Mangifera indica* L. and *Annona sp*., are also present on farms and in gardens [5]. The people of these villages depend on the Miombo forest not only for food, but also for the supply of wood and coal, that remain the most widely used fuels for cooking, brick making and woodworking. Other products that derive from the forest are the majority of natural fibers, for the production of ropes, baskets and rugs, and most of the medicinal plants [19].

### Selection of the informants and interview method

In general, ethnobotanical studies performed for cognitive and preservative purposes of local knowledge aim at collecting as much information as possible [24]. However, when conducting interviews, it is sometimes useful to select a preferencial topic to avoid wasting time, but above all, to investigate and preserve a specific and sometimes mostly endangered type of knowledge.

In the present study, beside the collection of general ethobotanical information, the attention was focused on plant medicinal use. The selection of candidates for interviews was performed with the collaboration of the Mazingira Association (http://www.mazingira.net/) that runs environmental education projects in the study area and operates in close contact with the resident population. Given the limited extension of the study area and the hesitancy of some local healers in sharing their knowledge, it was possible to interview only ten people.

The interviewes were carried out between June and August 2014, to ten candidates belonging to seven different tribal groups (Hehe, Pare, Chaga, Pogoro, Luguru, Mndamba, Mndegereko) with four people belonging to the Hehe group, predominant in this territory [12].

Each informant was interviewed individually for one or more times, according to his amount of knowledge. The interviews were always held in familiar places, to make it easier for the interwiewee to find the species with whom he/she is familiar. Each interview was conducted in Swahili language and mediated by an interpreter (Swahili-English) to allow the respondent to easily express his/her knowledge. For completeness and standardization of collected information, interviews were conducted by means of semi-structured questionnaires [25] (Additional file 1). The questionnaires were specifically developed for the purposes of the present study, based on a previous ethnobotanical research [26].

**Fig. 2:**
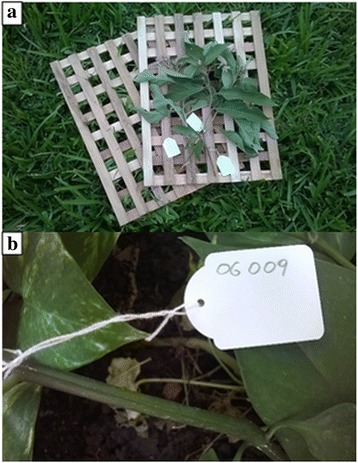
Plant sample collection and numbering. **a** *Solanum incana* specimen ready to be dried and pressed; **b** example of collection number

The interview consisted of three steps. At the first meeting, interviewer and informant were introduced to each other, the objectives of the study were explained in detail and the informed consent of the candidate to the interview, filming and taking photographs was acquired (Additional file 1). In a second phase, the personal information of the respondent, such as age, tribal group, profession and education level, was collected through a semi-structured questionnaire. Finally, the ethnobotanical knowledge was investigated. For each mentioned plant species, the informant was asked to provide information regarding the parts of the plant used, the method and period of harvesting, possible uses (e.g., food, fiber production, timber, spiritual uses, etc.) and whether the plant is still commonly used. Although a pre-structured questionnaire was used for the collection of this information, the candidate was left to speak freely and only at the end of the discussion specific questions were addressed to complete the data. Each interview was itinerant and took place under the guidance of the candidate, who was moving within the territory from which he/she usually collects plants, to directly show the plant species. The interviews usually took place in the morning before lunch and lasted between 3 and 4 h. After each interview, the candidate was payed 5000 Tsh (about 2.10 Euros) as compensation for the time spent. In addition, candidates received a paper, written in English and Swahili, with the purposes of the study to which they had just contributed. During the interviews, specimens were collected for an herbarium (Fig. 2a), after having acquired permission from the interviewee for plants located on his/her private property. Plant samples were never collected inside protected areas, therefore, when it was not possible to collect herbarium samples, detailed photographs of the plant species and of the used parts were taken.

All material was immediately tagged and marked by a progressive code of five numbers (Fig. 2b, Table 1): the first two digits indicate the respondent, while the following three digits identify the plant species. The plants (both in the form of herbarium samples and/or photographs) were firstly classified on the basis of diagnostic characters and validated by using the correspondence with the folk name provided by the informant (Table 1). All the plant samples were delivered to the Department of Botany of Dar-es-Salaam University where voucher specimens were collected and deposited. The plant species were classified following the standard botanical nomenclature according to The Plant List (http://www.theplantlist.org/).

**Table 1:** List of plants mentioned by informants in the study area and botanically classified with at least the botanical family name

Collection number	Family	Genus and species	Number of citations	Folk name	Growing habit	Wild\ cultivated	Main uses	Parts used	Cured diseases	Other uses
08003	Acanthaceae	*Hygrophila auriculata* (Schumach.) Heine	*	Mbigiri	herb	W	M	leaves	wounds	
03003	Amaranthaceae	*Amaranthus sp*.	*	Mchicha	herb	C	F	plant		
01005	Anacardiaceae	*Schinus sp.*	*	Mpilipili	tree	W	F	fruit		
08008	Anacardiaceae	*Sclerocarya birrea* (A.Rich.) Hochst	*	Mbwegere	tree	W	M	bark	gastrointestinal	
02009	Annonaceae	*Annona muricata* L.	*	Mstafeli	tree	C	F	fruit		
07012	Annonaceae	*Annona reticulata* L.	*	Topetope	tree	C	F	fruit		
01013	Apocynaceae	*Landolphia kirkii* Dyer	*	Kibanalomo	climber	W	F	fruit		
01020	Apocynaceae	*Saba comorensis* (Bojer ex A.DC.) Pichon	*	Lingombe	climber	W	F	fruit		
01033	Apocynaceae	*Tabernaemontana pachysiphon* Stapf	**	Mkomba, Mlowolowo	tree	W	M	roots, leaves	gastrointestinal	
01010	Apocynaceae	unidentified	*	Kihongola	shrub	W	R	branches		knives, handles
02015	Araceae	*Colocasia esculenta* (L.) Schott	*	Magimbaji	herb	C	F	roots, leaves		
02016	Araceae	*Colocasia sp.*	*	Magimblima	herb	C	F	roots		
05030	Araceae	*Philodendron sp.*	*	Mtambala panya	herb	W	M	leaves	respiratory	
02017	Arecaceae	*Elaeis guineensis* Jacq.	*	Mchikichi	palm	C	F	fruit		
01021	Arecaceae	*Phoenix reclinata* Jacq.	*	Ukindu	palm	W	R	leaves		baskets, mats
01039	Arecaceae	unidentified	*	Msalisi	palm	W	F	leaves		
01009	Asparagaceae	*Asparagus flagellaris* (Kunth) Baker	*	Mwinika	herb	W	F	plant		
08007	Bignoniaceae	*Kigelia africana* (Lam.) Benth	***	Mfungutua, Mfumbili	tree	W	M, O	bark, fruit	pain and inflammations, gastrointestinal	
03002	Brassicaceae	*Brassica sp.*	*	Chinese	herb	C	F, M	leaves	gynaecological, andrological and urinogenital	
03011	Brassicaceae	unidentified	*	Figiri, Leshuu	herb	C	F, M	leaves, plant	pain and inflammations, cardio-circulatory	
04015	Caricaceae	*Carica papaya* L.	*	Mpapai	tree	C	F	fruit		
01025	Cecropiaceae	*Myrianthus holstii* Engl.	*	Mfusa	tree	W	F	fruit		
01023	Clusiaceae	*Allanblackia stuhlmannii* (Engl.) Engl	*	Mkanyi	tree	W	F	fruit		
01003	Combretaceae	*Combretum sp.*	*	Mlama	tree	W	R	plant		charcoal, traditional beehives
06006	Combretaceae	*Terminalia catappa* L.	*	Mkungu	tree	C	F, R	fruit, plant		firewood
04006	Compositae	*Bidens pilosa* L.	*	Mashona nguo	herb	W	F	leaves		
05024	Compositae	*Emilia coccinea* (Sims) G.Don	*	Muelishi	herb	W	F	leaves		
01016	Compositae	*Lactuca inermis* Forssk.	**	Mchunga	herb	W	F	leaves		
01007	Compositae	unidentified	*	Jungujungu	herb	W	M	leaves	wounds	
05016	Compositae	unidentified	*	Mnyamgoha	herb	W	M	plant	gynaecological, andrological and urinogenital	
05023	Compositae	unidentified	*	Munosa	herb	W	M	flowers	eyes disesaes	
07004	Compositae	unidentified	*	Mganagana	herb	W	M	plant	typhus	
04003	Convolvulaceae	*Ipomoea batatas* (L.) Lam.	**	Kiazi kitam	herb	C	F	roots		
02010	Convolvulaceae	*Ipomoea sp.*	*	Matembele	herb	C	M, F	leaves	weakness and faints	
08001	Convolvulaceae	unidentified	*	Kaberega	herb	W	M	leaves	gynaecological, andrological and urinogenital	
02014	Crassulaceae	*Kalanchoe sp.*	*	Msharif	succulent	C	M	leaves	gastrointestinal, cardio-circulatory	
04005	Cucurbitaceae	*Cucurbita sp.*	*	Majani ya Maboga, Maboga	herb	C	F	leaves		
01029	Cucurbitaceae	*Momordica foetida* Schumach.	*	Delega	herb	W	F	leaves		
04013	Cucurbitaceae	*Telfairia pedata* (Sm.) Hook.	*	Mkweme	climber	C	F	seeds		
03005	Cyperaceae	unidentified	*	Makangaga	herb	W	R	plant		roof cover
02020	Dracaenaceae	*Dracaena fragrans* (L.) Ker Gawl.	*	Msae	shrub	C	O	plant		fence, decoration
01019	Dracaenaceae	*Dracaena mannii* Baker	*	Mshindamaji	shrub	W	M	leaves, bark	gastrointestinal, parasites, aphrodisiac	
01004	Ebenaceae	*Diospyros loureiroana* G.Don	*	Mdaa, Nyakatitu	shrub	W	O	plant		colorant
01035	Euphorbiaceae	*Bridelia micrantha* (Hochst.) Baill.	*	Mwiza	tree	W	R, O	log, bark		roof frames, colorant
01012	Euphorbiaceae	*Jatropha sp.*	*	Mtowo	shrub	W	R	bark		ropes and strings
02022	Euphorbiaceae	*Manihot esculenta* Crantz	***	Kisamvu	shrub	C	F	leaves, roots		
06002	Lamiaceae	*Gmelina arborea* Roxb.	*	Mfudufudu	tree	C	R	log		furniture
07011	Lamiaceae	*Leonotis nepetifolia* (L.) R.Br.	*	Kitengetenge	herb	C	M	leaves	malaria, hernia, typhus	
05040	Lamiaceae	*Ocimum gratissimum* L.	*	Mfumbeza	herb	W	M	plant	incontinence	
02013	Lamiaceae	*Ocimum tenuiflorum* L.	*	Mvumbasi, Mlumbasi	shrub	C	M	leaves	teeth an gums	insect repellent
02019	Lamiaceae	*Tectona grandis* L.f.	*	Mtiki	tree	C	R	log		furniture
04002	Lamiaceae	unidentified	*	Mwidu	herb	W	F	leaves		
05005	Lamiaceae	unidentified	*	Nyaka bondwa	herb	W	M	roots	snake bites	
05025	Lamiaceae	unidentified	*	Muhesita	herb	W	M	plant	children incontinence	
08005	Lamiaceae	unidentified	*	Mjekijeki	herb	W	M	leaves	wounds	
09012	Lamiaceae	unidentified	*	Uftapori	herb	W	M	leaves	malaria	
01040	Lamiaceae	*Vitex doniana* Sweet	****	Mcoga, Mfulu	tree	W	M, F, R	bark, leaves, fruits, log	weakness and faints	spoons, carved objects
09003	Leguminosae	*Abrus precatorius* L.	*	Ufambo, Kizunguzungu	climber	W	M	leaves	weakness and faints	
05010	Leguminosae	*Acacia sp.*	*	Mtalula	tree	W	M	leaves, roots	gynaecological, andrological and urinogenital	
07001	Leguminosae	*Acrocarpus fraxinifolius* Arn	*	Mkalati	tree	W	M	bark	gastrointestinal	
05038	Leguminosae	*Albizia schimperiana* Oliv.	*	Msere	tree	W	R	branches		handles
05039	Leguminosae	*Bauhinia petersiana* Bolle	**	Myegea, Msegese	tree	WC	M	bark	respiratory, diabetes	
03008	Leguminosae	*Brachystegia sp.*	*	Miombo	tree	W	R, O	plant, pollen		charcoal, bee plant
04008	Leguminosae	*Cajanus cajan* (L.) Millsp.	***	Mbaazi	shrub	C	M, F, R	leaves, seeds, plant	teeth and gums, otitis	firewood
05017	Leguminosae	*Crotalaria sp.*	*	Muvele	herb	W	M	leaves	headaches	
05012	Leguminosae	*Dalbergia melanoxylon* Guill. & Perr.	*	Mpingo, Mningo	tree	W	O	log, leaves		carved objects, furniture, ritual against snakes
01011	Leguminosae	*Entada sp.*	*	Lifute	climber	W	P, O	leaves, bark		strong detergent
01037	Leguminosae	*Erythrophleum suaveolens* (Guill. & Perr.) Brenan	*	Mbaraka, Muhehe	tree	W	R	log		furniture
09010	Leguminosae	*Piliostigma thonningii*	*	Msegete	tree	W	M	leaves	gastrointestinal, gynaecological, andrological and urinogenital	
01001	Leguminosae	*Pterocarpus angolensis* DC.	**	Mninga, Mnynga	tree	W	R, M	log, bark	gynaecological, andrological and urinogenital	furniture
05006	Leguminosae	*Senna siamea* (Lam.) H.S.Irwin & Barneby	*	Msonobari, Mjohoro	tree	WC	M, R	roots, plant		firewood
10003	Leguminosae	*Tamarindus indica* L.	**	Mkwaju	tree	WC	M, F	leaves, fruit	respiratory, gastrointestinal	parasites
01043	Leguminosae	unidentified	*	Likunde	herb	W	F	flowers		
04009	Leguminosae	unidentified	*	Linyala	shrub	W	F	leaves		
04014	Leguminosae	unidentified	*	Fiwi	climber	C	F	seeds		
05008	Leguminosae	unidentified	*	Mfuna	herb	W	M	plant	weakness and faints	
05015	Leguminosae	unidentified	*	Mugoba	tree	W	M	leaves	respiratory	
05029	Leguminosae	unidentified	*	Mkalangangumbi	herb	C	O	leaves		ritual to increase chicken growth
08006	Leguminosae	unidentified	*	Liwowo	shrub	W	F	leaves		
08010	Leguminosae	unidentified	*	Msawere	climber	W	M	roots	headaches	
09013	Leguminosae	unidentified	*	Limbatamba	shrub	W	M	roots, leaves	cardio-circulatory	
05046	Liliaceae	unidentified	*	Muheri	herb	W	M	plant	gastrointestinal	
01034	Logoniaceae	*Anthocleista grandiflora* Gilg	*	Mbala	tree	W	O	leaves		poisons
04012	Malvaceae	*Abelmoschus esculentus* (L.) Moench	*	Bamia	herb	C	F	fruits		
02021	Malvaceae	*Hibiscus sabdariffa* L.	*	Karkade	shrub	C	M	leaves, fruits	cardio-circulatory	
05019	Malvaceae	*Hibiscus surattensis* L.	***	Likakanapi, Mnyanyani	herb	W	M	leaves	eyes diseases, gastrointestinal	
02008	Meliaceae	*Cedrela odorata* L.	*	Msenderela	tree	C	R	log	gastrointestinal	furniture
02007	Meliaceae	*Khaya anthotheca* (Welw.) C.DC.	*	Mkangazi	tree	C	R	plant	anemia	furniture, firewood
06004	Mimosaceae	*Acacia auriculiformis* Benth.	*	Msegerea, Mzanzibari	tree	C	R	plant		furniture, firewood
01042	Mimosaceae	*Parkia filicoidea* Oliv.	*	Mnieze	tree	W	F, R	fruit, log		furniture, building materials
02003	Moraceae	*Artocarpus heterophyllus* Lam.	*	Fenesi	tree	C	F	fruits		
09011	Moraceae	*Ficus sycomorus* L.	*	Mkuyu	tree	W	M, O	bark, leaves	weakness and faints, wounds	ritual against evil eyes
05054	Moraceae	*Milicia excelsa* (Welw.) C.C.Berg	*	Mvule, Myange	tree	W	M, R	bark, log		furniture
01036	Moraceae	*Treculia africana* Decne. ex Trécul	*	Msaia	tree	W	F	fruits, seeds	weakness and faints	
02004	Moringaceae	*Moringa oleifera* Lam.	*	Mlonge	tree	C	M	leaves, seeds	malaria, weakness and faints	
03012	Musaceae	*Musa x paradisiaca* L.	*	Mgomba	herb	C	F, R	fruit, log		strings
06003	Myrtaceae	*Eucalyptus tereticornis* Sm.	**	Mlingoti, Mkalatusi	tree	C	R, M	log, bark, leaves	respiratory	poles
02006	Myrtaceae	*Psidium guajava* L.	**	Mpera	tree	C	M, F	leaves, fruit	gastrointestinal	
01027	Myrtaceae	*Syzygium guineense* (Willd.) DC.	*	Mzambarau, Mvenge	tree	WC	F	fruit		
05028	Nyctaginaceae	*Bougainvillea spectabilis* Willd.	*	Mpropes	shrub	C	M, O	leaves, flower, plant	parasites	decoration, acarus repellent
02005	Oxalidaceae	*Averrhoa bilimbi* L.	*	Mbilimbi	tree	C	F	fruit		
01017	Piperaceae	*Piper capense* L.f.	*	Likundukundu	shrub	W	F	fruit		
10008	Poaceae	*Cymbopogon citratus* (DC.) Stapf	*	Mchaichai	herb	C	F	leaves		poles
03004	Poaceae	*Oxytenanthera abyssinica* (A.Rich.) Munro	*	Mianzi ya ulanzi	herb	C	F, R	sap, log		poles
03006	Poaceae	unidentified	*	Magugu	herb	W	R	plant		roof cover
03007	Poaceae	unidentified	*	Magugu	herb	W	R	plant		roof cover
01014	Rubiaceae	*Uncaria africana* G.Don	*	Likamanda	climber	W	F	plant		
02024	Rutaceae	*Citrus limon* (L.) Osbeck	*	Lemon tree	tree	C	F	fruit		
02018	Rutaceae	*Citrus nobilis* Lour.	*	Tangerine	tree	C	F	fruit		
01041	Sapotaceae	*Synsepalum msolo* (Engl.) T.D.Penn.	*	Msambisa	tree	W	F, R	fruit, log		poles and handles
03001	Solanaceae	*Capsicum chinense* Jacq.	**	Mpilipili	herb	C	F, O	fruit		repellent
10004	Solanaceae	*Nicotiana tabacum* L.	*	Tumbaco	herb	C	O	leaves		cigars
07009	Solanaceae	*Physalis peruviana* L.	*	Songosongo	herb	C	M	leaves	respiratory	
01018	Solanaceae	*Solanum incanum* L.	**	Mdulele, Mtula	shrub	W	M	fruit, roots	parasites, bites, gastrointestinal	
06001	Solanaceae	*Solanum melongena* L.	*	Ngogwe, Nyanyachungu	herb	C	F	fruit		
03010	Solanaceae	*Solanum sp.*	*	Mnafu	herb	C	F	leaves		
02023	Sterculiaceae	*Theobroma cacao* L.	*	Cacao	tree	C	F	fruit, seeds		
05026	Verbenaceae	*Duranta erecta* L.	*	Msekela	shrub	C	O	leaves, roots		ritual

Frequency: * one citation, ** two citations, *** three citations, **** four citations

*C* cultivated species, *W* wild species, *WC* both wild and cultivated species

*M* medicinal use, *F* food use, *R* production of raw material, *O* other uses

Despite all efforts, 78 plant species could not be classified given the lack of samples and sometimes of diagnostic characters in the specimens. The unidentified plants are listed in the Additional file 2 using just the vernacular name given by the informant. Even tough the interviewees exactly described the medical proprieties and other uses of these plant species, they were not included in the following data processing.

## Results and discussion

### Informants

In total ten informants were interviewed, three women and seven men, aged between 31 and 86 years, with a mean age of 53 and a median of 53. Six respondents were over 50 years old. Ethnobotanical knowledge was not equally shared between the two genders and the average number of quoted species was 13.7 for men and 7.3 for women. On average, male informants reported 5.8 food plants and female 5.6. Similarly, the number of medicinal species cited was 5.7 for males and 6.0 for females. This reflects the fact that traditional ethnobotanical knowledge equally passed on through both the male and female line.

The informants were traditional healers as first or second job and all practiced farming. The respondents belonged to different ethnic groups: four were of the Hehe tribe, the dominant tribal group in the investigated area, while the other six candidates were one each of the Pare, Chaga, Pogoro, Luguru, Mndamba, Mndegereko tribes. On average education level was low, with nine candidates having attended just primary school and only one who also attended secondary school.

### Data on plant species

Overall 196 different plant species traditionally used in the four villages of the study area were mentioned. For 118 samples, at least the family and, in most cases, the genus and species were idientified (Table 1), while for 78 plants only the folk name was available despite the efforts of classifying them (Additional file 2). Taking into consideration similar researches conducted in study areas neigbouring the Kilombero valley, the percentage of identified species (Table 1) in common with previous studies was 15.3% respect to those detected by Amri et al. [5], 20.3% respect to Shangali et al. [19] and 5.9% respect to Chrispin et al. [20].

**Fig. 3:**
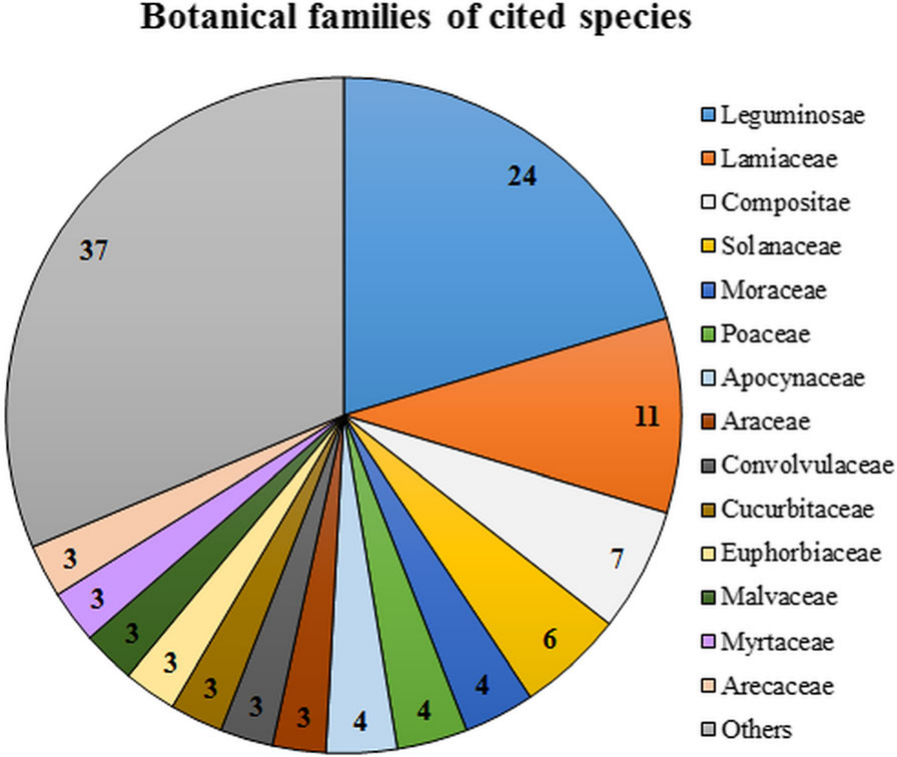
Main botanical families of plant species mentioned by informants in the Kilombero Valley

Most of the plant species belonged to the family of Leguminosae (24), followed by Lamiaceae (11), Compositae (7) and Solanaceae (6) (Fig. 3). This result is in agreement with similar studies carried out in the Morogoro region [18] and other areas of Tanzania, where Leguminosae, Compositae and Solanaceae were among the most aboundant families [13, 16, 18]. In comparison with previous studies [13, 16, 18], the present data revealed a large presence of Lamiaceae, probably due to the widespread coltivation of this plant family (e.g., *Ocimum tenuiflorum* L., *O. gratissimum* L.) around the villages. Data evidenced the absence of plants belonging to the Rubiaceae family, as instead previously detected in other studies in the Morogoro region [18]; this was most probably due to the lack of forest areas nearby the studied villages and to the fact that most plants were collected in grassland and shrub areas, where this family is rarely present. Of all recorded species, 38.1% were trees, 38.1% herbs, 13.6% shrubs, 6.8% climbers, 2.5% palms and 0.8% succulent plants, which is again in accordance with previous studies which detected a large prevalence of tree species [5, 13]. Of the 118 identified species (Table 1), 67 were collected in the wild, 47 were cultivated and 4 were both cultivated and spontaneous, such as *Tamarindus indica* L. Overall, 52% of the reported useful species were exotic plants, while the remaining 48% were native plants.

Ethnobotanical knowledge is very diverse among the various tribes and for this reason multiple citations of plants by different healers are usually rare [12] (Table 1). The most cited plant is *Vitex doniana* Sweet (by 4 respondents), commonly used to prepare a tonic and energizing infusion. *Manihot esculenta* Crantz, *Kigelia africana* (Lam.) Benth., *Cajanus cajan* (L.) Millsp. and *Hibiscus surattensis* L. were cited by 3 respondents, but while the first is only used as food, the other three species are used for many purposes, including food, fuels, medicine and rituals (Table 1).

### Uses of cited species

**Fig. 4:**
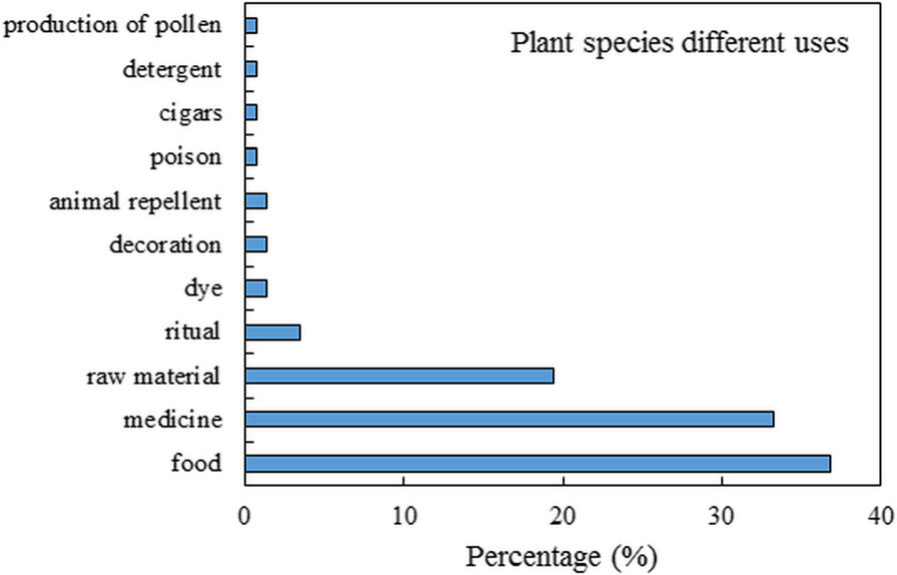
Different uses of the plant species mentioned by informants and botanically classified in the study area

Most of the identified species were used for food purpose (36.8%), and medicinal use (33.3%), followed by production of raw materials, such as wood and fibers (19.4%), and ritual use (3.5%) (Fig. 4). When considering the totality of the mentioned species (196, Table 1 plus Additional file 2), the medicinal species represented the majority (45%), which is in agreement with other studies carried out in the Morogoro region [5].

Of the identified plants (Table 1), 79.7% have only one use, 18.6% have two uses (e.g., *Psidium guajava* L., used as food and medicine) and 1.6% have three different uses (e.g., *C. cajan* L. Millsp, used as medicine, food and fuel). Some of the mentioned plants (14 species among which *V. doniana*, *Synsepalum msolo* (Engl.) T.D.Penn., *Parkia filicoidea* Oliv. *and Milicia excelsa* (Welw.) C.C.Berg) are highly valued trees, not only as food (for their edible fruits) and medicines, but also as firewood and timber. This fact is causing a fast depletion of the number of individuals of these wild species due to the indiscriminate cutting down of the trees.

### Medicinal use

**Fig. 5:**
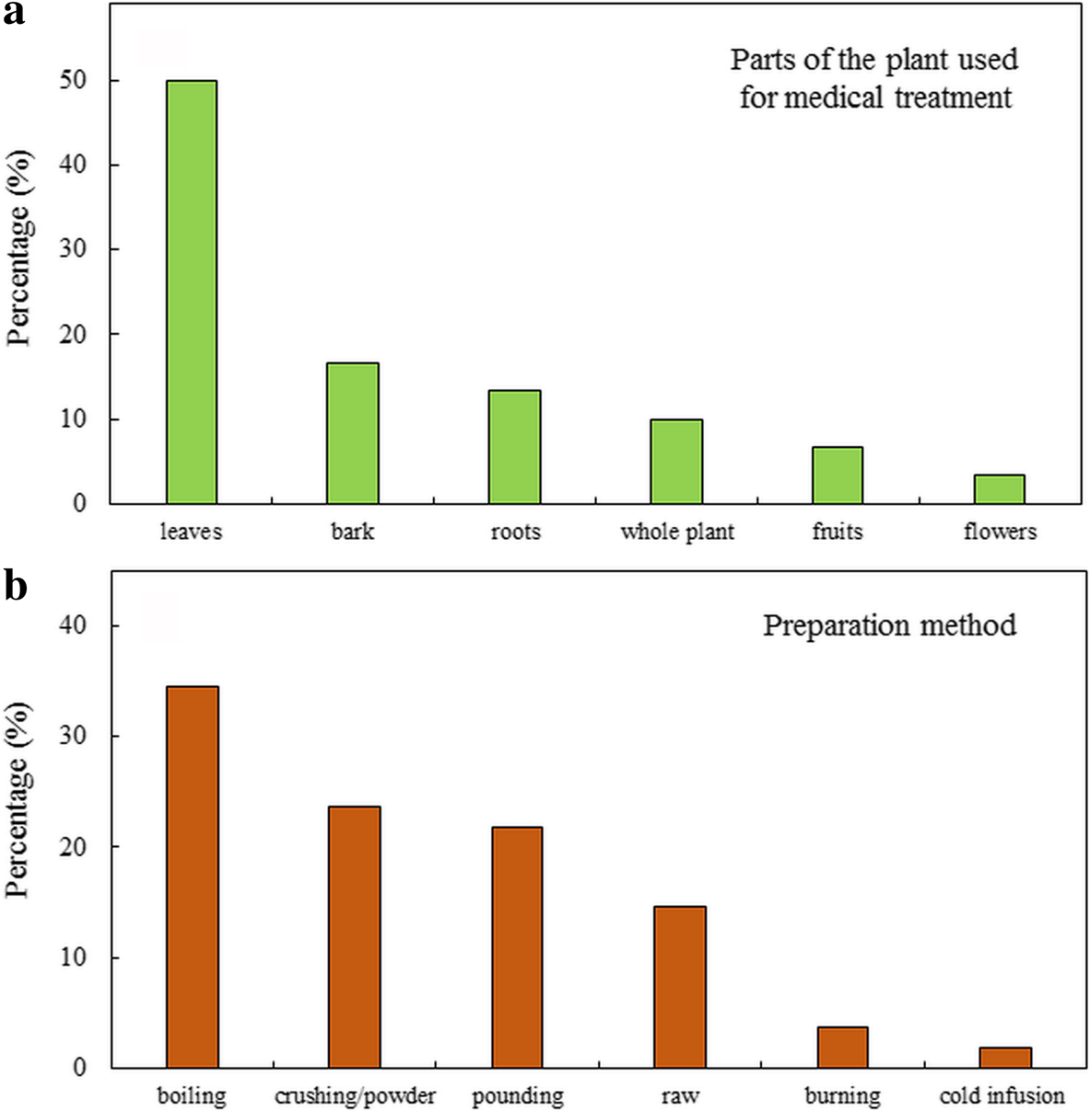
Plant species for medical treatments. **a** Used parts of the plants; **b** preparation methods

**Fig. 6:**
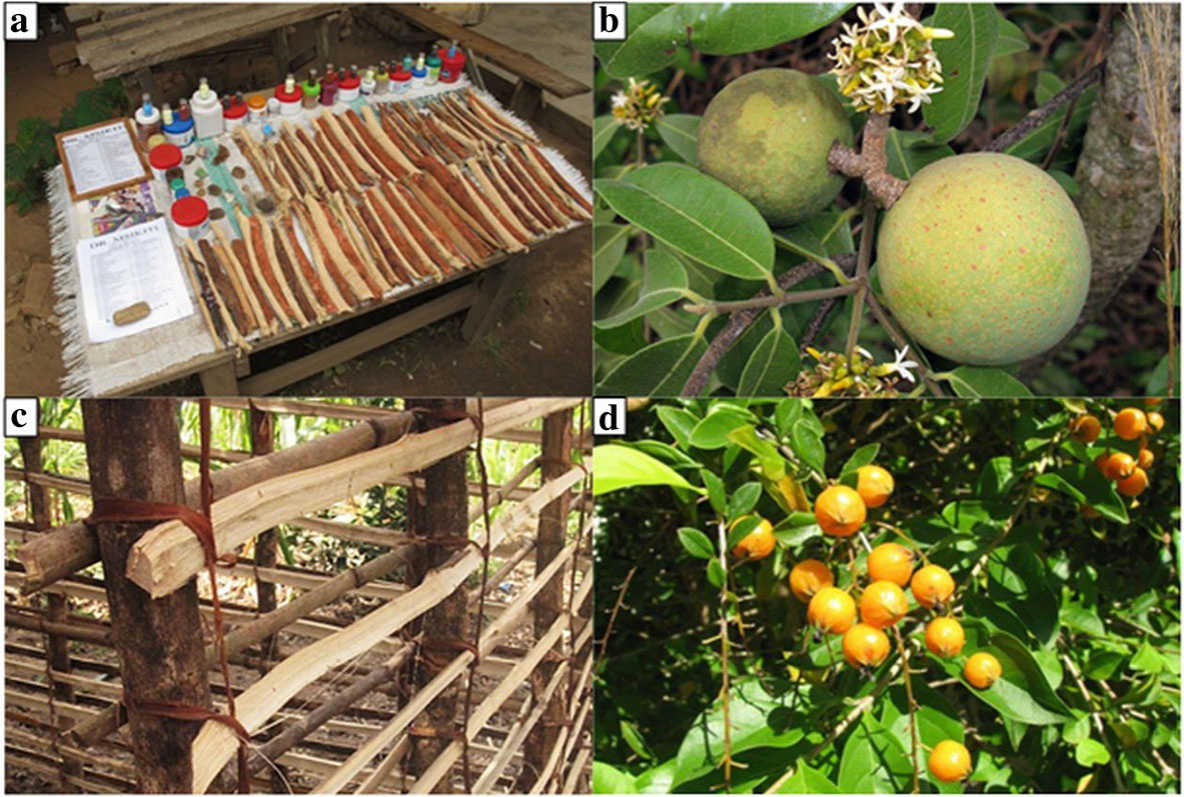
Plant different uses. **a** Stand of a local healer with numerous barks for sale; **b** fruits of *Landolphia kirkii* Dyer, a wild liana; **c** the bark of *Brachystegia sp.* used to tie together the elements of traditional houses; **d** *Duranta erecta* L., a ritual species, used against misfortune

The parts of the plants (Fig. 5a) mainly used for the preparation of herbal remedies were leaves (50%), bark (16.7%, Fig. 6a), roots (13.3%), whole plant (10%), fruits (6.7%) and other parts like sap or flowers (3.3%). A similar distribution was found by other studies in Africa [5, 13, 16, 18], with in general leaves representing the most frequently used plant part for medicine. Conversely, other studies reported roots as the most used part in preparing drugs [12, 27].

Most of the medicinal plants mentioned by the informants were usually harvested in the wild (32 species), while only few were cultivated (13 species). Therefore, plant harvesting methods have a deep impact on the natural ecosystem. In fact, while the collection of leaves somehow preserves the integrity of the plant, the excavation of roots and decortication, have extremely deleterious effects on plant individuals leading to their premature death. According to the opinion of the informants, many of the medicinal species subjected to such practices are at present seriously threatened and are becoming increasingly rare in their habitats. This triggers a vicious cycle of an even larger exploitation of the remaing specimens, which will accelerate their disappearance as already reported by Amri et al. [5] in other Morogoro region sites.

In 71.4% of cases, the mentioned medicinal plants were indicated for the treatment of a single disease, 22.5% of the species were used to cure two diseases, while the remaining 6.1% were used in the treatment of three or more diseases (such as *Dracaena mannii* Baker, *Leonotis nepetifolia* (L.) R.Br., *Solanum incanum* L.). Among the most cured diseases were gastrointestinal pathologies (diarrhea, vomit, stomach ache) cured by 11 species, weakness and faints (7 species), gynaecological, andrological and urinogenital disorders (erection, infertility, bleeding losses) cured by 7 species, respiratory diseases (cough, cold) (6 species), parasites (6 species), cardio-circulatory problems (4 species) and wounds (3 species) (Table 2). Other studies showed intestinal pathologies [5] and also wounds, respiratoty, urinogenital and cardio-circulatory (e.g. hypertension) disorders as those most frequently treated with medicinal plants [18].

**Table 2:** Type of diseases cured by plants botanically identified in the area of study (see Table 1)

Disease	Number of species suitable for treatment
Gastrointestinal	11
Weakness and faints	7
Gynaecological, andrological and urinogenital	7
Respiratory	6
Parasites	6
Cardio-circulatory	4
Wounds	3
Typhus	2
Pain and inflammations	2
Teeth and gums	2
Incontinence	2
Bites	2
Headaches	2
Eye problems	2
Diabetes	1
Hernia	1
Otitis	1
Anemia	1

Medicinal treatments were most commonly prepared by boiling the plant part containing the active substance (34.5%), by crushing the dried part of the plant (23.6%), or by pounding the fresh plant parts, (21.8%), whereas sometimes the collected plant organs were used raw (18%) (Fig. 5b). These results are in accordance with investigantions carried out in the same region [5] and in general with other ethnobotanical studies in Tanzania, in which boiling to make decotions and pounding/grinding resulted to be the most common medicine preparation methods [13, 18]. Medical treatments were assumed by ingestion (77.4%), by dermal application (20.8%) and by inhalation (1.9%). Considering also the unclassified species (Additional file 2), other ritual methods can be found, such as the dispersion on the ground of the drug.

It should be taken into account that many of the traditional healing methods are based on rituals, dreams and spirit evocations (typical of every local healer), which most of the times are believed to be more important than the actual effect of plant medicinal remedies. This further diversifies the picture of traditional medicine, which largely varies from tribe to tribe, according to their own tradition.

### Food and other plant uses

In total 53 plants were also indicated for food use: 21 were species collected in the wild, 30 were cultivated and 2 were both cultivated or grow spontaneously in the wild. Most of these belong to the Fabaceae family (6 species), followed by Solanaceae, Asteraceae and Cucurbitaceae (3 species each). The parts of the plants mostly consumed as food were fruits and seeds (55.6%), leaves (31.5%), roots (7.4%) and shoots (5.6%). Wild food plants do not play a key role in the diet of these communities and are not commonly used as famine foods. According to the present data, wild fruits such as *Landolphia kirkii* Dyer or *Vitex doniana* Sweet fruits, are collected as snacks by people working in the fields; wild vegetables such as *Bidens pilosa* L., *Lactuca inermis* Forssk. or *Asparagus flagellaris* (Kunth) Baker are used as side dishes; other species like *Piper capense* L.f. are added as flavours to some main dishes.

In general, fruits were eaten raw, while vegetables were consumed cooked in 80% of cases, while the remaining 20% was consumed raw or used for infusion to extract the aromas, such as *Cymbopogon citratus* (DC.) Stapf. Among species commonly collected in the wild there were *Bidens pilosa*, an herb used as a vegetable, and the fruits of *Landolphia kirkii* Dyer (Fig. 6b). Instead, *Telfairia pedata* (Sm.) Hook., a native cultivated species for collecting seeds (in order to extract oil), was indicated as progressively disappearing due to its low productivity compared to the new species of oil plant recently introduced.

Twenty-eight (19.4%) species were indicated as useful to produce raw materials (Fig. 4), of which 21 were woody species used for the production of furniture, house structural elements, firewood and poles. The other seven species were herbs (e.g., *Poaceae and Cyperaceae*) and palms which are mainly used for the production of fibers and roof covers (Fig. 6c). One of the most exploited species is *Dalbergia melanoxylon* Guill. & Perr. used because of its dark wood to make carved objects, such as figurines and necklaces. *Phoenix reclinata* Jacq. and *Musa x paradisiaca* L. were mainly used for the production of cordage and for weaving works as reported in Shangali et al. [19]

In addition, five other species (Fig. 4) were indicated as having ritual purposes linked to their traditional use within the communities. These species were thought to avoid bad luck (e.g., by drinking an infusion of *Duranta erecta* L. leaves and roots for 21 days, Fig. 6d), or against bad sprits or evil eye (e.g., bark of *Ficus sycomorus* L.). As also reported in [19], 2 plants were used to extract dyes (*Bridelia micrantha* (Hochst.) Baill. and *Diospyros loureiroana* G.Don). In the house gardens many species were just used as decoration, among which the most frequently cited by respondents were *Dracaena fragrans*, (L.) Ker Gawl. *Bougainvillea spectabilis* Willd. and *Duranta erecta* L. One wild tree (*Brachystegia sp.*) was indicated as pollen producer for bees, while the leaves of the wild poisonous tree *Anthocleista grandiflora* Gilg were used by local fishermens to stun fishes as also reported also by Shangali et al. [19]. Even in these rural community, as well as worldwide, *Nicotiana tabacum* L. leaves were used for the production of cigars. Finally, *Capsicum chinense* Jacq., *Ocimum tenuiflorum* L. and *Bougainvillea spectabilis* were indicated to be good repellent for insects (Fig. 4).

## Conclusions

The present study revealed that numerous plant species are still essential in the everyday life of the tribes living in Kilombero Valley. These plants are commonly used for medicinal, food, weaving and building purposes. Most of the plants mentioned by the interviewed people were usually harvested in the wild, threatening the existence of some useful species. After the creation of the Udzungwa Mountains National Park, the collection areas were highly reduced concentrating the harvesting pressure on the few remaining areas of unprotected forest. Only few healers started to cultivate species for disease treatment, while almost all started to collect the plants in the neighbouring natural environment. Present data point out that half of the medicinal remedies were prepared from leaves (50%), while 16.7% were obtained from bark, 13.3% from roots, and 10% from the whole herbaceous plant. Harvesting practices like root excavation and stem decortication are causing a progressive depletion of many medicinal plant species. In addition, deforestation makes medicinal species harvesting areas increasingly scarce, forcing many local healers to abandon the practice. In the light of these facts, it is essential, in the immediate future, to educate traditional healers as well as common people to the sustainable use of the surrounding natural heritage. It seems also necessary to provide the populations with additional means to increase the forested areas, such as the distribution of seedlings for biomass production. Although some efforts have already been made in the studied territory, and in spite of a firm tradition in Tanzania of community-based forest conservation, the situation remains critical and the state of unprotected forests near these villages is deteriorating year after year. This situation, if not quickly reversed, may lead to an unprecedented environmental crisis and to the loss of much of the traditional ethnobotanical culture. In this context, the present study wishes to contribute, at least to some exent, to preserving the knowledge present in the investigated populations, still deeply connected to nature, and to passing down this unevaluable tradition to future generations.

## Additional files

Additional file 1: Questionnaire form used during the semi-striuctured interviews of the ethnobotanical survey. (PDF 271 kb)
Additional file 2: List of plants not classified mentioned in the study area. (PDF 97 kb)

## References

[1] Cotton CM (1996). Ethnobotany: principles and applications.

[2] Abbiw DK (1990). Useful plants of Ghana: West African uses of wild and cultivated plants.

[3] Kochhar SL (1998). Economic botany in the tropics.

[4] Cunningham AB. Applied ethnobotany: people, wild plant use and conservation. People and plants conservation manuals. London: Earthscan Publications Ltd. and VA USA: Sterling; 2001.

[5] Amri E, Kisangau DP (2012). Ethnomedicinal study of plants used in villages around Kimboza forest reserve in Morogoro, Tanzania. J Ethnobiol Ethnomed.

[6] Le Roux PJ (1981). Supply of fuel-wood for rural populations in South Africa. South African Forestry J.

[7] The World Bank. http://data.worldbank.org/indicator/SP.RUR.TOTL.ZS.

[8] World Health Organization. Traditional medicine. Fact sheet No 134; 2003. http://www.who.int/en/.

[9] Kisangau DP, Lyaruu HV, Hosea KM, Joseph CC (2007). Use of traditional medicines in the management of HIV/AIDS opportunistic infections in Tanzania: a case in the Bukoba rural district. J Ethnobiol Ethnomed.

[10] Rural Poverty Portal. http://www.ruralpovertyportal.org/country/home/tags/Tanzania.

[11] Mahunnah RLA, Usio FC, Kayombo EJ (2012). Documentary of traditional medicine in Tanzania.

[12] Gessler MC, Msuya DE, Nkunya MHH, Mwasumbi LB, Schär A, Heinrich M, Tanner M (1995). Traditional healers in Tanzania: the treatment of malaria with plant remedies. J Ethnopharmacol.

[13] Moshi MJ, Donald F, Otieno R, Mbabazi PK, Weisheit A (2010). Ethnomedicine of the Kagera Region, Northwestern Tanzania. Part 2: the medicinal plants used in Katoro ward, Bukoba District. J Ethnobiol Ethnomed.

[14] Hall J, Burgess ND, Lovett J, Mbilinyi B, Gereau RE (2009). Conservation implications of deforestation across an elevational gradient in the Eastern Arc Mountains, Tanzania. Biol Conserv.

[15] Liengme CL (1983). A study of wood use for fuel and building in an area of Gazankulu. Bothalia.

[16] Moshi MJ, Otieno DF, Mbabazi PK, Weisheit A (2009). The ethnomedicine of the Haya people of Bugabo ward, Kagera Region, Northwestern Tanzania. J Ethnobiol Ethnomed.

[17] Schlage C, Mabula C, Mahunnah RLA, Heinrich M (2000). Medicinal plants of the Washambaa (Tanzania): documentation and ethnopharmacological evaluation. Plant Biol.

[18] Moshi MJ, Otieno DF, Weisheit A (2012). Ethnomedicine of the Kagera Region, Northwestern Tanzania. Part 3: plants used in traditional medicine in Kikuku village, Muleba District. J Ethnobiol Ethnomed.

[19] Shangali CF, Mabula CK, Mmari C (1998). Biodiversity and human activities in the Udzungwa Mountain Forest, Tanzania. Ethnobotanical survey in the Udzungwa Scarp Forest Reserve. J East Af Nat Hist.

[20] Shangali CF, Zilihona IJE, Mwang’ingo PLP, Nummelin M (2008). Use of medicinal plants in the Eastern Arc Mountains with special reference to the Hehe ethnic group in the Udzungwa Mountains, Tanzania. J East Af Nat Hist.

[21] Luoga EJ, Witkowski ET, Balkwill K (2000). Differential utilization and ethnobotany of trees in Kitulaghalo forest reserve and surrounding communal lands, Eastern Tanzania. Econ Bot.

[22] McGowan PJK, Dowell SD, Carroll JP, Aebischer NJ (1995). Partridges, quails, francolins, snowcocks and guineafowl: status survey and conservation action plan 1995–1999.

[23] Frost P, Campbell B (1996). The ecology of miombo woodlands. The Miombo in transition: woodlands and welfare in Africa.

[24] Signorini MA, Bruschi P, Camangi F, Guarrera PM, Pieroni A, Savo V, Caneva G, Pieroni A, Guarrera PM (2013). Metodi della ricerca etnobotanica. Etnobotanica. Conservazione di una patrimonio culturale come risorsa per uno sviluppo sostenibile.

[25] Alexiandes MN, Alexiandes MN (1996). Collecting ethnobotanical data: an introduction to basic concepts and techniques. Selected guidelines for ethnobotanical research: a field manual.

[26] Sebastiani L, Camangi F, Stefani A (2009). Plant biodiversity, recovery, and conservation: a case study from the val di Vara (La Spezia - Liguria). Acta Hortic.

[27] Chalabra SC, Mahunnah RLA, Mshiu EN (1993). Plants used in traditional medicine in Eastern Tanzania. VI. Angiosperms (Sapotaceae to Zingiberaceae). J Ethnopharmacol.

