# Supplementary material for: Traditional knowledge on wild and cultivated plants in the Kilombero Valley (Morogoro Region, Tanzania)
Source: J Ethnobiol Ethnomed. 2017 Mar 9;13:17. doi: 10.1186/s13002-017-0146-y (PMC5345176; doi:10.1186/s13002-017-0146-y)
Supplement: Additional file 1: — Questionnaire form used during the semi-striuctured interviews of the ethnobotanical survey. (PDF 271 kb) [file 13002_2017_146_MOESM1_ESM.pdf]

Ethnobotanical data collecting sheet

**Informant code** _ _	**Date:**

1. Informants' consent for the participation in the study:

Mimi.. (jina la mhusika), kwa hiari yangu na akili zangu timamu bila kulazimishwa, nimeamua kushiriki katika utafiti huu na ninaahidi kuwa kwa ulewa wangu wote, maelezo niliyoyatoa ni ya kweli. sahihi na yaliyo kamilika.
Tarehe..Saini..

2. Informant details:

Name:

Gender: **M** **F**

Age:

Ethnic group:

Occupation:

Education:

Ethnobotanical expertise (*where/how the informant learned the expertise*):

Are you teaching it?

3. Location details:

Name of the village:

Region\ district:

Gps coordinate:

Altitude:

4. Notes:

**Informant code:** _ _	**Date:**

1. Plant details:

Collection number: (informant code + plant number) _ _ _ _ _

Collector name:

Plant local common name: Language:

Meaning of the local name:

Scientific name:

Family:	
Genus:	Species:

2. Collected:

Herbarium specimen:

yes	no	N. of specimens:

Seeds:

yes	no

Photos:

yes	no	Files:

3. Plant description:

herb	shrub	tree	bulb	tuber	climbing	other:

Other characteristics:

**Wild species**	**Cultivated species**

Ecology:

Availability

Common	Found in some areas	Rare

Common	Seasonal product	Rare

Availability on the market of plants/ seeds/ products:

Abundance respect the past:

Uses:	Plant part used:
Medicine	
Food	
Fodder	
Poison	
Raw material	

4. Cultivation (only for cultivated species):

Propagation methods:

Seeds	Cuttings	Division	Other:

Irrigation:

Fertilization:

Light requirement:

Pest and disease / control:

Others particular coltural cures:

5. Harvest:

Method and period of harvesting:

Storage:

6. Details of use for medicinal plants:

Disease(s) treated:

Method of drug preparation:

Treatment (way of assumption, dosage, frequency):

Side effects:

7. Details of food uses:

Method of food preparation:

8. Raw material:

Products

Method of preparation

9. Other uses:
