# Supplementary material for: Traditional knowledge on wild and cultivated plants in the Kilombero Valley (Morogoro Region, Tanzania)
Source: J Ethnobiol Ethnomed. 2017 Mar 9;13:17. doi: 10.1186/s13002-017-0146-y (PMC5345176; doi:10.1186/s13002-017-0146-y)
Supplement: Additional file 2: — List of plants not classified mentioned in the study area. (PDF 97 kb) [file 13002_2017_146_MOESM2_ESM.pdf]

Additional file 2. List of plants mentioned by informants in the study area and not botanically classified.

Collection number	Family	Genus and species	Number of citations	Folk name	Growing habits	wild\ cultivated	Main uses	Parts used	Cured diseases	Other uses
01002			*	Mpingo	tree	W	R	log		sculpture, necklaces
01006			*	Memenagolo	tree	W	R	branches		poles
01008			*	Mlyafiosi	herb	W	R	log		hornamental sticks
01015			*	Finunu	climber	W	F	fruit		
01022			*	Mkongo	tree	W	R	log		furniture
01024			*	Mkelegeti	tree	W	F	fruit		
01026			*	Kisani	shrub	W	R	branches		shephard sticks
01028			*	Mpande, Mkwaju	tree	W,C	F	fruit		
01030			*	Nalufumbula	herb	W	M	leaves	nightmares	
01031			*	Lupusa	tree	W	R	branches		arrows
01032			*	Likamanda	climber	W	M	sap	wounds	
01038			*	Sunguluti	tree	W	R	branches		chairs
01044			*	Mulimuli	tree	W	F	fruit		
01045			*	Pembaduma	tree	W	M	leaves, roots	hernia	
01046			*	Mandala	tree	W	O	seeds		ritual against eyes diseases
01047			*	Lulemambale	climber	W	R	bark		ropes
02001			*	Kitundawe	tree	C	M,O	leaves, roots	nightmares	house protection against witches

02002		*	Mauwa sumu	succulent	W,C	O	sap, plant		termites control, decoration, poison
02011		*	Mpiripisi,	tree	C	F	fruit		
05001		*	Msungula	shrub	W	M	leaves, roots	wounds	
05002		*	Msungula, Mdogo	shrub	W	M	leaves	pain and inflammations	
05003		*	Nyawahe	herb	W	M	roots, leaves	wounds	
05007		*	Mhumbahumba	shrub	W	M	roots, leaves	malaria	
05009		*	Mkwambekwambe	shrub	W	M	plant	pain and inflammations, gynaecological, andrological and urinogenital	
05011		*	Msheli	herb	W,C	O	plant		snake protection, decoration
05013		*	Msangalasi	tree	W	O	roots, leaves		treatment effect increaser
05014		*	mnepa	tree	W	R,O	log, leaves		furnitures, love potion
05018		*	Munyilava	herb	W	M	leaves	fever	
05020		*	Mungwasi	shrub	W	M	leaves	pain and inflammations	
05021		*	Muswavi	herb	W	M	plant	pain and inflammations	
05022		*	Macho ya mlungu	climber	W	M, O	leaves, fruit	aphrodisiac	against evil eyes,

05027	*	Mnygi	herb	W	M	plant	animal disease	
05031	*	Mzilinga	herb	W	M	leaves	weakness and faints	
05032	*	Mwija, Mwiza	tree	W	R	bark		colorant
05033	*	Mgoweko	tree	W	M	leaves, bark	pain and inflammations	
05034	*	Mninga, Muvembadanda	tree	W	M	bark	gynaecological, andrological and urinogenital	
05035	*	Mugundutati	tree	W	M	leaves	gynaecological, andrological and urinogenital	
05036	*	Mtundufulu	herb	W	O	plant		harvesting ritual
05037	*	Muhafue	tree	W	M	leaves	weakness and faints	
05041	*	Mwesefu	shrub	W	O	leaves		mosquito repellent
05042	*	Mulaganzuki	tree	W	O	flowers		bees attractor
05043	*	Mwija	tree	W	M	leaves	animal disease	
05044	*	Mdomongo	tree	W	M	sap	wounds	
05045	*	Mwitosi	herb	W	M	roots	respiratory	
05047	*	Mweli	tree	W	O	leaves		against evil spirits
05048	*	Mtunumbi	tree	W	M	leaves	weakness and faints	
05049	*	Mukwangwasale	climber	W	M	roots	parasites	
05050	*	Mndukule	herb	W	M	roots	bites	

05051	*	Mfagio	herb	W	R	plant		brooms
05052	*	Mbodogo	tree	W	M	leaves	animal disease	
05055	*	Nyakatitu	tree	W	M	leaves	bones fractures	
05056	*	Mmemena	tree	W	M	leaves	eyes diseases	
05057	*	Mjendiko	shrub	W	M	roots, leaves	cardio-circulatory	
06005	*	Miashoki	tree	C	O	plant		decoration
07005	*	Mtopetope	tree	W	M	bark, leaves	eyes diseases respratory	
07006	*	Muvembadanda	tree	W	M	roots, leaves	gastrointestinal	
07007	*	Mchichapori	herb	W	M	leaves	gastrointestinal	
07008	*	Nyawahe	herb	W	M	leaves	gastrointestinal	
07010	*	Limoyo	tree	W	M	roots	cardio-circulatory	
07013	*		tree	W	M	leaves	weakness and faints	
07016	*	Mtogo	tree	W	M	bark	gynaecological, andrological and urinogenital	
07017	*	Mpingipingi	tree	W	M	bark	gynaecological, andrological and urinogenital	
08002	*	Kikwambikwambi	shrub	W	M	leaves	gynaecological, andrological and urinogenital	
08009	*	Sugusugu	tree	W	M	roots	gastrointestinal	

09001	*	Mkombajiko	tree	W	M	leaves, roots	gynaecological, andrological and urinogenital	
09002	*	Msada	climber	W	O	leaves		wild animal repellent
09006	*	Mtutuma	tree	W	M	roots	parasites	
09007	*	Mkwambekwambe	tree	W	M	roots	pain and inflammations	
09008	*	Mtopetope	tree	W	M	bark, leaves	parasites, gastrointestinal	
09014	*	Luchici	herb	W	M	roots	headache	
09015	*		herb	W	M,O	leaves	otitis, hemorrhoids	against evil spirits
09016	*		climber	W	M	leaves	weakness and faints	
09017	*	Msolwa	tree	W	O	branches		ritual against thieves
10001	*	Madelapori	herb	W	M	leaves	hemorrhoids	
10002	*	Mnunganunga, Mavimavi	herb	W	M,O	leaves	malaria	against evil spirits
10005	*	Mderega	herb	C	F	leaves		
10007	*	Embegonogo	tree	C	F	fruit		
10009	*	Ina	shrub	C	O	leaves		nails colorant

Frequency: * one citation; ** two citations; ***: three citations; **** four citations.
C: cultivated species, W: wild species, WC: both wild and cultivated species.
M: medicinal use, F: food use, R: production of raw material, O: other uses
